# Streptomycetes: Attractive Hosts for Recombinant Protein Production

**DOI:** 10.3389/fmicb.2020.01958

**Published:** 2020-08-20

**Authors:** Francesca Berini, Flavia Marinelli, Elisa Binda

**Affiliations:** Department of Biotechnology and Life Sciences, University of Insubria, Varese, Italy

**Keywords:** streptomycetes, recombinant proteins, heterologous expression, industrial enzymes, therapeutic proteins

## Abstract

Enzymes are increasingly applied as biocatalysts for fulfilling industrial needs in a variety of applications and there is a bursting of interest for novel therapeutic proteins. Consequently, developing appropriate expression platforms for efficiently producing such recombinant proteins represents a crucial challenge. It is nowadays widely accepted that an ideal ‘universal microbial host’ for heterologous protein expression does not exist. Indeed, the first-choice microbes, as *Escherichia coli* or yeasts, possess known intrinsic limitations that inevitably restrict their applications. In this scenario, bacteria belonging to the *Streptomyces* genus need to be considered with more attention as promising, alternative, and versatile platforms for recombinant protein production. This is due to their peculiar features, first-of-all their natural attitude to secrete proteins in the extracellular milieu. Additionally, streptomycetes are considered robust and scalable industrial strains and a wide range of tools for their genetic manipulation is nowadays available. This mini-review includes an overview of recombinant protein production in streptomycetes, covering nearly 100 cases of heterologous proteins expressed in these Gram-positives from the 1980s to December 2019. We investigated homologous sources, heterologous hosts, and molecular tools (promoters/vectors/signal peptides) used for the expression of these recombinant proteins. We reported on their final cellular localization and yield. Thus, this analysis might represent a useful source of information, showing pros and cons of using streptomycetes as platform for recombinant protein production and paving the way for their more extensive use in future as alternative heterologous hosts.

## Introduction

Nowadays, we witness the increasing application of enzymes in industrial sectors, including food, detergent, and textile manufactures (Trono, 2019) and the bursting of interest in proteins for therapeutic and diagnostic purposes (Tripathi and Shrivastava, 2019). Developing efficient bioprocessing strategies for protein production is consequently of utmost importance. Most of valuable industrial enzymes and therapeutic proteins are recombinant versions, produced by heterologous platforms (Adrio and Demain, 2014). However, an ideal ‘universal host’ for protein heterologous expression does not exist. Those microbes (as *Escherichia coli* or yeasts) that are still considered the first-choices to this purpose possess intrinsic limitations inevitably restricting their use. Production of heterologous proteins in *E. coli* is limited by self-cytotoxicity, incorrect folding, aggregation into inclusion bodies, and/or lack of secretion (Adrio and Demain, 2014). In yeasts, recombinant protein production is often associated with hyper-glycosylation and product retention within the periplasmic space (Vieira Gomes et al., 2018).

**FIGURE 1:**
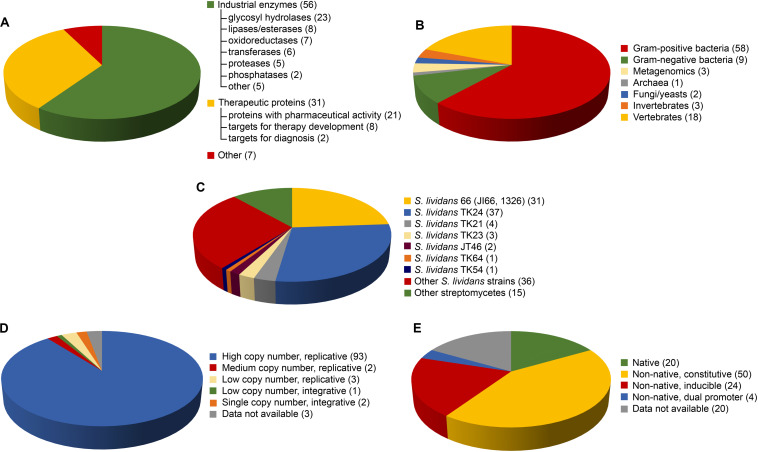
Distribution of the 94 recombinant proteins heterologously produced by streptomycetes and listed in Table 1, according to: use/family of the recombinant protein **(A)**, homologous source **(B)**, heterologous host **(C)**, plasmid **(D)**, and promoter **(E)** used for heterologous production. **(C**–**E)** When for the same protein, multiple hosts and/or plasmids, and/or promoters were used, these different conditions were counted separately.

In this scenario, bacteria belonging to the *Streptomyces* genus might represent a promising alternative platform for recombinant protein production. Streptomycetes are Gram-positive, aerobic bacteria, characterized by a mycelial life style and commonly found in soils, where they secrete multiple hydrolytic enzymes to degrade complex organic substrates. This natural secretion capacity represents their most attractive feature for recombinant protein production. Secretion may prevent local accumulation of the overexpressed recombinant proteins, reducing toxicity to host cells and promoting correct folding (Anné et al., 2012). It facilitates downstream recovery decreasing production costs (Hamed et al., 2018). In addition, streptomycetes are characterized by low endogenous proteolytic activity; they grow relatively fast and in inexpensive media; they do not produce pyrogenic lipopolysaccharides and endotoxins; they are not pathogenic; and they might express G + C-rich genes without codon usage optimization (Anné et al., 2012; Sevillano et al., 2013). Thanks to the extensive fermentation knowhow deriving from their use as antibiotic producers (Ndlovu et al., 2015), streptomycetes are robust and scalable industrial strains, and a wide range of tools for their genetic manipulation have recently become available (Kieser et al., 2000). Notwithstanding these potential advantages, nowadays their use is not so common as it could be expected. To investigate this aspect, in this mini-review we cover – to the best of our knowledge – all studies published from 1980s to December 2019, in which streptomycetes were used as heterologous hosts for recombinant protein production. Table 1 reports these 94 cases of proteins expressed in streptomycetes. Figure 1 highlights the main results emerging from the analysis of Table 1 in terms of protein class, homologous source, heterologous host, and molecular tools.

**TABLE 1:** List of the heterologous proteins produced by streptomycetes (in chronological order).

References	Protein	Source	Heterologous host	Plasmid	Promoter	Signal peptide	Productivity (up to)	Localization
Berini et al., 2019	Chitinase	Metagenomics	*S. coelicolor* A3(2), *S. venezuelae* ATCC 10595, *S. lividans* TK24	pIJ86	*ermE*p*	Absent	45 mg/L	Extracellular
Cavaletti et al., 2019	Glutenase	*Actinoallomurus* sp. (Gram +)	*S. lividans* TK24	pIJ86	*ermE*p*	Native	1.4 × 10^6^ U/L	Extracellular
Šnajder et al., 2019	Pernisine	*Aeropyrum pernix* (archaeon)	*S. rimosus* M4018	pVF	*tcp830*p	*srT*-SP	10 mg/L (codon usage optimization, pro-region removal)	Extracellular
Tao et al., 2019	Phospholipase D	*S. halstedii* (Gram +)	*S. lividans* TK24	pIJ12739	Dual promoter (*tipA*p/*ermE*p*)	Native	7.1 × 10^4^ U/L	Extracellular
Carrillo Rincón et al., 2018	Phytase	*Escherichia coli* (Gram −)	*S. rimosus* M4018	pVF, pAB04	*ermE*p*, nitA/nitRp, *tcp830*p	*aml*-SP_*Sv*_, *srT*-SP, *lip*-SP	5 × 10^3^ U/L in extracellular fraction, < 1 × 10^3^ U/L in cytoplasm (codon usage optimization)	Extracellular + cytoplasm
Daniels et al., 2018	Cellulase	*Rhodothermus marinus* (Gram −)	*S. lividans* TK24	pIJ486	*vsi*p	*vsi*-SP	7.5 mg/L	Extracellular
Noguchi et al., 2018	Chitobiase	*S. avermitilis* (Gram +)	*S. lividans* 1326 and derivative (expressing a repressor to avoid protein production without inducer)	pIJ350	*xylA*p_*Sa*_	Native	1.5 × 10^6^ U/L	Extracellular
Hamed et al., 2017	Cellulase	*Rhodothermus marinus* (Gram −)	*S. lividans* TK24	pIJ486	*vsi*p	*vsi*-SP	90 mg/L (120 mg/g dry cell weight)	Extracellular
Sevillano et al., 2017	α-Amylase	*S. griseus* (Gram +)	*S. lividans*Δ*TA-Tox* (pGM160-YefMsl^*ts*^, pALCre^*ts*^)	pNRoxAnti	*pstS*p	NA	1.1 × 10^6^ U/L	Extracellular
	Xylanase	*S. halstedii* (Gram +)				NA	1.7 × 10^5^ U/L	Extracellular
Gabarró et al., 2016	Agarase	*S. coelicolor* (Gram +)	*S. lividans* TK21, *S. lividans*Δ*sipY* (derivative deficient in the major signal peptidase SipY)	pIJ486	Native	NA	2.4 × 10^6^ U/L	Extracellular
	Laccase	*S. lividans* (Gram +)	*S. lividans*Δ*sipY* (derivative deficient in the major signal peptidase SipY)	pFD666	*dag*p	NA	5.8 U/L	Extracellular
Liu et al., 2016	Transglutaminase	*S. hygroscopicus* (Gram +)	*S. lividans* TK24	pIJ86	Native (optimized by removal of negative element)	Native	5.7 × 10^3^ U/L (codon usage optimization)	Extracellular
Nguyen-Thi and Doucet, 2016	Chitinase	*S. coelicolor* (Gram +)	*S. lividans* 10-164	pC109	NA	NA	1.1 × 10^3^ mg/L	Extracellular
Sevillano et al., 2016	Xylanase	*S. halstedii* (Gram +)	*S. lividans* 1326, *S. lividans* GSAL1 (derivative overexpressing the morphogene *ssgA*)	Derivative of pN702GEM3	Native, *vsi*p, *ermE*p*, *xysA*p, *pstS*p, *xylA*p_*Sc*_, *glpQ*p	Native, *amy*-SP (as-it-is, or optimized)	2.5 × 10^5^ U/L	Extracellular
	α-Amylase	*S. griseus* (Gram +)			*xysA*p, *pstS*p	Native	1.6 × 10^5^ U/L	Extracellular
	Laccase	*S. coelicolor* (Gram +)	*S. lividans* 1326, *S. lividans*Δ*xlnR, S. lividans*Δ*bxlR* (derivatives knocked-out in putative *xysA*p repressor genes)	pHJL401	*xysA*p	Native	160 U/g dry weight	Extracellular
Guan et al., 2015	Transglutaminase	*S. hygroscopicus* (Gram +)	*S. lividans* TK24, *S. griseus, S. lividans* 1326, *S. hygroscopicus* FR008	pIJ86	Native, *ermE*p	Native (as-it-is, or optimized)	687 mg/L (9.6 × 10^3^ U/L)	Extracellular
	Aminopeptidase	*Bacillus subtilis* (Gram +)			*tg*p	*tg*-SP (optimized)	2.8 × 10^3^ U/L	Extracellular
	Phenylalanine ammonia-lyase	*Rhodotorula glutinis* (yeast)					2.1 × 10^4^ U/L	Extracellular
Gullón et al., 2015	Agarase	*S. coelicolor* (Gram +)	*S. lividans* TK21, *S. lividans*Δ*secG, S. lividans*Δ*tatC* (derivatives knocked-out for components of the Sec- or Tat-route respectively)	pAGAs1	Native	Native, *aml*-SP_*Sl*_	60 U/mg dry weight	Extracellular
Torres-Bacete et al., 2015	Penicillin V acylase	*S. lavendulae* (Gram +)	*S. lividans* 1326	pEM4	*ermE*p*	Native	11 mg/L (959 U/L)	Extracellular
Binda et al., 2013	D,D-peptidase/D,D-carboxypeptidase	*Nonomuraea gerenzanensis* (Gram +)	*S. venezuelae* ATCC 10595, *S. coelicolor* A3(2), *S. lividans* TK24	pIJ86	*ermE*p*	Native	12 mg/L	Cell wall fraction
Li et al., 2013	Endoglucanase	*Thermobifida fusca* (Gram +)	*S. lividans* 1326	pZRJ362	*xylA*p_*Am*_	Native	173 mg/L (5.6 × 10^3^ U/L)	Extracellular
Sevillano et al., 2013	α-Amylase	*S. griseus* (Gram +)	*S. lividans* pKC796, *S. lividans*Δ*TA-pKC796* (pGM160- YefMsl^*ts*^), *S. lividans*Δ*TA-pKC796-Tox* (pGM160-YefMsl^*ts*^)	pN702Gem3-Anti	*pstS*p	NA	NA	Extracellular
	Xylanase	*S. halstedii* (Gram +)				NA	NA	Extracellular
Lule et al., 2012	Tumor Necrosis Factor α	Human	*S. lividans* TK24 and derivative (overexpressing phosphoenolpyruvate carboxykinase)	pIJ486	*vsi*p	*vsi*-SP	0.9 mg/g dry biomass	Extracellular
Dubeau et al., 2011	Chitosanase	*Kitasatospora* sp. N106 (Gram +)	*S. lividans* TK24, *S. lividans*Δ*csnR* (knocked-out for a negative transcriptional regulator)	Derivative of pHM8a, pFDES	Native (as-it-is or modified), *S. ghanaensis* phage I19 promoter	NA	2.4 × 10^4^ U/L	Extracellular
Nakazawa et al., 2011	Phospholipase D	*S. racemochromo genes* (Gram +)	*S. lividans* TK23	pES	Native	NA	3.0 × 10^4^ U/L	Extracellular
Zhu et al., 2011	Interleukin A	Human	*S. lividans* TK24	Derivative of pSGL1	*ermE*p*	*melC1*-SP, *gpp*-SP (as-it-is, or optimized)	0.6 mg/L	Extracellular
Côté and Shareck, 2010	Lipase	Metagenomics	*S. lividans* 10-164	pIAFC109	NA	Native	NA	Extracellular
Noda et al., 2010	Transglutaminase	*Stv. cinnamoneum* (Gram +)	*S. lividans* 1326	pIJ702	*plD*p	*plD*-SP	230 mg/L	Extracellular
	β-1,4-Endoglucanase	*Thermobifida fusca* (Gram +)					64 mg/L	Extracellular
	β-Glucosidase						114 mg/L	Extracellular
Sinsereekul et al., 2010	Cutinase	*Thermobifida* sp. (Gram +)	*S. rimosus* R7	pIJ8600	*tipA*p	Native	58 μg/L	Extracellular
Meilleur et al., 2009	Lipase	Metagenomics	*S. lividans* IAF10-164	pIAFD95A	*D95A*p	Native	11.3 mg/L	Extracellular
Díaz et al., 2008	Alkaline phosphatase	*Thermus thermophiles* (Gram −)	*S. lividans* JI66	pIJ702	*xysA*p	Native	2.7 × 10^5^ U/L	Extracellular
	β-Glycosidase					Absent	2.6 × 10^5^ U/L in cytoplasm, 5.5 × 10^4^ U/L in extracellular fraction	Extracellular + cytoplasm
Dubé et al., 2008	Laccase	*S. coelicolor* (Gram +)	*S. lividans* IAF10-164	pIAFD95A	*D95A*p	NA	350 mg/L	Extracellular
Hatanaka et al., 2008	Leucine aminopeptidase	*S. griseus* (Gram +)	*S. lividans* 1326	pTONA5	*ssm*p, *ermE*p*, kibilysin gene promoter	NA	1.5 × 10^5^ U/L	Extracellular
	Proline aminopeptidase	*Streptomyces* sp. (Gram +)				Absent	5.2 10^5^ U/L in extracellular fraction, 5.0 × 10^4^ U/L in cytoplasm	Extracellular + cytoplasm
	Aminopeptidase P					Absent	3.5 × 10^4^ U/L in extracellular fraction, up to 1.8 × 10^4^ U/L in cytoplasm	Extracellular + cytoplasm
Lin et al., 2006, 2008	Tranglutaminase	*S. platensis* (Gram +)	*S. lividans* JT46	pIJ702	*melC1*p	Native	5.8 × 10^3^ U/L	Extracellular
Qi et al., 2008	Glucagon (co-expressed with rat α-amidase gene)	Human	*S. lividans* TK24	Derivative of pIJ680	*aph*p	*melC1*-SP	24 mg/L	Extracellular
Ayadi et al., 2007	α-Integrin A-domain	Rat	*S. lividans* 1326	pIJ699	*ermE*p	Long synthetic SP	8 mg/L	Extracellular
Merkens et al., 2007	Quercetinase	*Streptomyces* sp. (Gram +)	*S. lividans* TK23	pIJ702	Native	Absent	5.1 U/mg total protein	Cytoplasm
Pimienta et al., 2007	Streptokinase	*Streptococcus equisimilis* (Gram +)	*S. lividans* TK24	pUWL-218	*vsi*p	*vsi*-SP, *xlnC*-SP	15 mg/L	Extracellular
Vrancken et al., 2007	Tumor Necrosis Factor α	Human	*S. lividans* TK24 and derivative (over-expressing the phage-shock protein A homolog)	pSSV05	*vsi*p	*vsi*-SP	1.1 μg/mg dry weight	Extracellular
	Enhanced green fluorescent protein	*Aequorea victoria* (jellyfish)				*xlnC*-SP	20 mg/L (15.9 U/mg dry weight)	Extracellular
Côté et al., 2006	β-Glucosaminidase	*Amycolatopsis orientalis* (Gram +)	*S. lividans* TK24	pFD666	NA	Native	573 U/L	Extracellular
	β-Glucosaminidase	*S. avermitilis* (Gram +)			NA	NA	NA	Extracellular
Sianidis et al., 2006	Xyloglucanase	*Jonesia* sp. (Gram +)	*S. lividans* TK24	pIJ486	*vsi*p	Native, *vsi*-SP	150 mg/L	Extracellular
Vallin et al., 2006	Glycoprotein (antigen)	*Mycobacterium tuberculosis* (Gram +)	*S. lividans* 1326	pUWL-219	*dag*p	*dag*-SP	80 mg/L	Extracellular
Fukatsu et al., 2005	N-substituted formamide deformylase	*Arthrobacter pascens* (Gram +)	*S. lividans* TK24, *S. coelicolor* A3(2) M145, *S. avermitilis* K139	pSH19	*nitA/nitR*p	NA	8.5 U/mg total protein	Extracellular
Rose et al., 2005	Latex clearing protein	*Streptomyces* sp. (Gram +)	*S. lividans* TK23	pIJ702	Native	Native	NA	Extracellular
Díaz et al., 2004	Xylanase	*Aspergillus nidulans* (fungus)	*S. lividans* JI66	pIJ702	*xysA*p	Native, *xys1*-SP	1.9 × 10^4^ U/L	Extracellular
Lara et al., 2004	Glycoprotein (antigen)	*Mycobacterium tuberculosis* (Gram +)	*S. lividans* 1326	pIJ486, pIJ6021	Native, *tipA*p	Native	5 mg/L	Extracellular
Lin et al., 2004	Transglutaminase	*Stv. ladakanum* (Gram +)	*S. lividans* JT46	pIJ702	Native	Native	1.5 × 10^3^ U/L	Extracellular
Ogino et al., 2004	Phospholipase D	*Stv cinnamoneum* (Gram +)	*S. lividans* 1326	pUC702	Native	Native	118 mg/L (5.5 × 10^4^ U/L)	Extracellular
Schaerlaekens et al., 2004	Tumor Necrosis Factor α	Human	*S. lividans* TK24, *S. lividans*Δ*tatB, S. lividans*Δ*tatC* (derivatives knocked-out for components of the Tat pathway)	pIJ486	*vsi*p	*xlnC*-SP, *melC1*-SP, *vsi*-SP	23 mg/L	Extracellular
	Interleukin-10						166 μg/L	Extracellular
Zhang et al., 2004	Interleukin-4 receptor	Human	*S. lividans* TK24	pSGLgpp	NA	*gpp*-SP	10 mg/L	Extracellular
Béki et al., 2003	β-D-Mannosidase	*Thermobifida fusca* (Gram +)	*S. lividans* TK24	pIJ699	Native	Absent	0.015 U/mg total protein	Cytoplasm
Geueke and Hummel, 2003	L-Amino acid oxidase	*Rhodococcus opacus* (Gram +)	*S. lividans* 1326	pIJ6021, pUWL201	*tipA*p, *ermE*p*	Native	18 U/L	Cytoplasm
Hong et al., 2003	Calcitonin (co-expressed with rat α-amidase gene)	Salmon	*S. lividans* TK54	pIJ680	*aph*p	*melC1*-SP	30 mg/L	Extracellular
Tremblay et al., 2002	19 kDa major lipoprotein antigens	*Mycobacterium tuberculosis* (Gram +)	*S. lividans* IA F10-164	pIJ702	*xlnA*p	*celA*-SP (long)	200 mg/L	Extracellular
	38 kDa major lipoprotein antigens						80 mg/L	Extracellular
Lammertyn et al., 1997; Pozidis et al., 2001	Tumor Necrosis Factor α	*Mus musculus* (Mouse)	*S. lividans* TK24	pIJ486	*vsi*p	*vsi*-SP (as-it-is or modified)	300 mg/L	Extracellular
Isiegas et al., 1999	β-Lactamase	*Escherichia coli* (Gram −)	*S. lividans* TK21	pIJ487	*dag*p	*dag*-SP	60 U/L	Extracellular
Smith et al., 1999	Alkene monooxygenase	*Rhodococcus rhodochrous* (Gram +)	*S. lividans* TK24	pIJ6021	*tipA*p	NA	2.2 U/mg total protein	Cytoplasm
Lammertyn et al., 1998	Tumor Necrosis Factor α	*Mus musculus* (Mouse)	*S. lividans*	pIJ486	*vsi*p	*aml*-SP_*Sv*_	50 mg/L	Extracellular
Park and Lee, 1998	β-Lactamase-inhibitory protein	*S. exfoliatus* (Gram +)	*S. lividans* TK24	pIJ702	*melC1*p	Native	3.0 × 10^4^ U/L	Extracellular
Binnie et al., 1997	Extracellular domain of erythropoietin receptor	Human	*S. lividans* 66	pCAN46	*aph*p	*sprtB*-SP (modified)	15 mg/L	Extracellular
Motamedi et al., 1996	31-*O*-Demethyl-FK506 methyltransferase	*S. hygroscopicus* (Gram +)	*S. lividans*	pIJ943	NA	Native	NA	Cytoplasm
Taguchi et al., 1995	Transforming Growth Factor α (fused with *S. albogriseolus* subtilisin inhibitor)	Human	*S. lividans* 66	pIJ702	*ssi*p + *melC1*p	*ssi*-SP	10 mg/L	Extracellular
Paradkar et al., 1994	β-Lactamase inhibitor protein	*S. clavuligerus* (Gram +)	*S. lividans* TK24	pIJ486	Native	Native	NA	Extracellular
Washizu et al., 1994	Transglutaminase	*Stv. mobaraense* (Gram +)	*S. lividans* 3131	pIJ702	*S. antibioticus* tyrosinase promoter	Native	0.1 mg/L	Extracellular
Fornwald et al., 1993	T cell receptor CD4 (as-it-is and derivatives)	Human	*S. lividans* 1326	pLTI450	*STI-II*p, β*gal*p	*STI-II*-SP	300 mg/L	Extracellular
Jung et al., 1993	Endoglucanase	*Thermobifida fusca* (Gram +)	*S. lividans* TK24	Derivatives of pIJ702	Native	Native	36 mg/L (1.9 × 10^3^ U/L)	Extracellular
	Exoglucanase				Native	Native	17 mg/L (23 U/L)	Extracellular
Ueda et al., 1993	Fv domain of monoclonal antibody against hen egg-white lysozyme	Human	*S. lividans* 66	pIJ702	*ssi*p	*ssi*-SP	1 mg/L	Extracellular
Wolfframm et al., 1993	Chloroperoxidase	*Pseudomonas pyrrocinia* (Gram −)	*S. lividans* TK64	pIJ486	Native	NA	11.2 U/g wet weight	Cytoplasm
Hale et al., 1992	Esterase	*S. scabiae* (Gram +)	*S. lividans* 1326	pIJ486, pIJ702	NA	Native	100 mg/L	Extracellular
Taguchi et al., 1992	Apidaecin 1b (fused with *S. albogriseolus* subtilisin inhibitor)	*Apis mellifera* (Honeybee)	*S. lividans* 66	pIJ702	*ssi*p + *melC1*p	*ssi*-SP	>200 mg/L	Extracellular
Jørgensen et al., 1991	Lipase (co-expressed with a lipase modulator)	*Pseudomonas cepacia* (Gram −)	*S. lividans* TK24	pIJ702	*dag*p	*dag*-SP	Na	NA
Bender et al., 1990a	Hirudin	*Hirudo medicinalis* (Leech)	*S. lividans* TK24	pIJ702	*melC1*p	*AI*-SP	500 μg/L	Extracellular
Bender et al., 1990b	Interleukin-2	Human	*S. lividans* TK24	pIJ680	NA	*AI*-SP	7.1 × 10^5^ U/L in extracellular fraction, 4.7 × 10^4^ U/L in cytoplasm	Extracellular + cytoplasm
Koller and Riess, 1989	Human α-amylase inhibitor (tendamistat)	*S. tendae* (Gram +)	*S. lividans* TK24	pIJ61, pIJ350, pIJ486, pIJ702	Native, *melC1*p (or both in tandem)	Native	700 mg/L	Extracellular
Swan et al., 1989	Calcium-binding protein	*Sac. erythraea* (Gram +)	*S. lividans* TK24	pIJ702	Native	NA	NA	Extracellular
Lamb et al., 1988	65-kilodalton antigen	*Mycobacterium leprae* (Gram +)	*S. lividans*	pIJ697	Native	NA	NA	Cytoplasm
Lichenstein et al., 1988	Interleukin-1β	Human	*S. lividans* 1326	pIJ350	β*gal*p	β*gal*-SP	3.8 × 10^6^ U/L in extracellular fraction, 6.3 × 10^4^ U/L in cytoplasm	Extracellular + cytoplasm
	Galaktokinase	*Escherichia coli* (Gram −)	*S. lividans* 1326, *S. lividans galK*^–^ (galactokinase-deficient mutant)				345 U/L in extracellular fraction, 120 U/L in cytoplasm	Extracellular + cytoplasm
Noack et al., 1988	Interferon α1	Human	*S. lividans* TK24	pIJ487	*saK*p	*saK*-SP	2.0 × 10^8^ U/L	Extracellular
Horinouchi et al., 1987	Streptothricin acetyltransferase	*S. lavendulae* (Gram +)	*S. lividans* TK21	pIJ41, pIJ702, pIJ487	*aph*p, *melC1*p, *Bacillus subtilis* cellulose promoter	NA	NA	Cytoplasm

*The list was created by searching Pubmed database (accession on 18 December, 2019) with the following query: ((((heterologous[Title/Abstract]) AND expression[Title/Abstract]) AND protein[Title/Abstract]) AND streptomyces[Title/Abstract]), then manually checked and integrated. Gram +, Gram-positive; Gram −, Gram-negative; NA, data not available; SP, signal peptide; S., Streptomyces; Sac., Saccharopolyspora; Stv., Streptoverticillium. Promoters (CO, constitutive; IN, inducible): aphp from S. fradiae aminoglycoside 3′-phosphotransferase (CO); βgalp from S. lividans β-galactosidase (CO); D95Ap from S. coelicolor groEL2 heat-shock gene (NA); dagp from S. coelicolor agarase (CO); ermEp^∗^ from Sac. erythraea erythromycin resistance gene (CO); glpQp from S. coelicolor glycerophosphoryl diester phosphodiesterase (IN by glycerol-3-phosphate); melC1p from S. antibioticus melanin operon (CO); nitA/nitRp from Rhodococcus rhodochrous nitrilase (IN by ε-caprolactam); plDp from Stv. cinnamoneum phospholipase D (CO); pstSp from S. lividans phosphate-binding protein (IN by phosphate starvation and carbon sources as fructose, xylose, or galactose); saKp from Staphylococcus aureus phage 42D staphylokinase (NA); STI-IIp from S. longisporus protease inhibitor (NA); ssip from S. albogriseolus subtilisin inhibitor (CO); ssmp from S. cinnamoneus metalloendopeptidase (CO in the presence of a rich inorganic phosphate source and glucose); tcp830p synthetic promoter from S. coelicolor (IN by tetracycline); tgp from S. hygroscopicus transglutaminase (CO); tipAp from S. lividans (IN by thiostrepton); vsip from S. venezuelae subtilisin inhibitor (CO); xlnAp from S. lividans xylanase A (NA); xylAp from S. avermitilis (xylAp_Sa_), S. coelicolor (xylAp_Sc_), or Actinoplanes missouriensis (xylAp_Am_) xylose isomerase (IN by xylose); xysAp from S. halstedii xylanase (IN by carbon sources as xylose, xylan, or fructose). Plasmids (HN, high copy number; MN, moderate copy number; LN, low copy number; SN, single copy number; int, integrative; rep, replicative): pAB04 (LN, int); pAGAs1 (HN, rep); pC109 (HN, rep); pCAN46 (HN, rep); pEM4 (HN, rep); pES (HN, rep); pFD666 (HN, rep); pFDES (HN, rep); pHJL401 (MN, rep); pHM8 (SN, int); pIAFC109 (HN, rep); pIJ12739 (MN, rep); pIJ350 (HN, rep); pIJ41 (LN, rep); pIJ486 (HN, rep); pIJ487 (HN, rep); pIJ6021 (HN, rep); pIJ61 (LN, rep); pIJ680 (HN, rep); pIJ699 (HN, rep); pIJ702 (HN, rep); pIJ86 (HN, rep); pIJ8600 (SN, int); pIJ943 (LN, rep); pLTI450 (HN, rep); pN702GEM3 (HN, rep); pN702Gem3-Anti (HN, rep); pNRoxAnti (HN, rep); pSGL1 (HN, rep); pSGLgpp (HN, rep); pSH19 (HN, rep); pSSV05 (HN, rep); pTONA5 (HN, rep); pUC702 (HN, rep); pUWL201 (HN, rep); pUWL-218 (HN, rep); pUWL-219 (HN, rep); pVF (HN, rep); pZRJ362 (HN, rep). Signal peptide: AI-SP from S. tendae tendamistat (α-amylase inhibitor); aml-SP from S. venezuelae (aml-SP_Sv_) or S. lividans (aml-SP_Sl_) α-amylase; amy-SP from S. griseus α-amylase; βgal-SP from S. lividans (β-galactosidase); celA-SP from S. lividans cellulase; dag-SP from S. coelicolor agarase; gpp-SP from S. globisporus apoprotein C-1027; lip-SP from S. rimosus lipase; melC1-SP from S. antibioticus melanin operon gene; plD-SP from Stv. cinnamoneum phospholipase D; saK-SP from Staphylococcus aureus phage 42D staphylokinase; sprtB-SP from S. griseus protease B; srT-SP from S. rimosus trypsin-like protease; ssi-SP from S. albogriseolus subtilisin inhibitor; STI-II-SP from S. longisporus protease inhibitor; tg-SP from S. hygroscopicus transglutaminase; vsi-SP from S. venezuelae subtilisin inhibitor; xlnC-SP from S. lividans xylanase C; xys1-SP from S. halstedii xylanase.*

## What Are the Recombinant Proteins Produced in Streptomycetes?

50 (out of 94) proteins listed in Table 1 are enzymes with potential industrial/environmental applications (Figure 1A). The most represented class is that of glycosyl hydrolases (23 proteins), including: (i) (hemi)cellulases, for lignocellulose saccharification and biofuel production; (ii) chitinases, for generating value-added chitin-derivatives as chitosan or biopesticides (Berini et al., 2018a); and (iii) amylases for starch processing. The lipase/esterase group (8 proteins) with applications in detergent, food, and biofuel industries, and the oxidoreductase class (7), including laccases and peroxidases for bioremediation (Berini et al., 2018b), follow. Interesting examples are the phospholipase D from *Streptomyces racemochromogenes*, for producing phosphatidyl derivatives from lecithin with emulsifying properties for food and cosmetics (Nakazawa et al., 2011), and the cutinase from *Thermobifida* sp. with polyester-degrading activity in bioplastic recycle (Sinsereekul et al., 2010). Dubé et al. (2008) produced in *Streptomyces lividans* up to 350 mg/L of *Streptomyces coelicolor* small laccase, a thermostable enzyme decolorizing synthetic dyes that is considered promising for pollutant degradation in urban or industrial wastewaters. Finally, Table 1 and Figure 1A include transferases (6 proteins) for food processing, proteases/peptidases (5) for feed and detergent industries, and phosphatases (2), including a phytase used as supplement for animal nutrition (Carrillo Rincón et al., 2018). Additionally, Torres-Bacete et al. (2015) expressed a novel Penicillin V acylase for producing semisynthetic penicillins, whereas Rose et al. (2005) a latex clearing protein for bioconversion of rubber wastes. Unfortunately, only few of these studies reported a comparison of protein expression yield between streptomycetes and other microbial hosts. Hamed et al. (2017) succeed in producing 90 mg/L of a thermostable cellulase from the bacteroidetes *Rhodotermus marinus* using *S*. *lividans* TK24 as host; the same protein could not be produced in *E. coli.* Very recently, Šnajder et al. (2019) reported the first and so far the only case of expression of an archaeal thermozyme (pernisine) in *Streptomyces rimosus*. The homologous host – the hyperthermophilic *Aeropyrum pernix* – was uncultivable in industrial fermentation facilities. The protein productivity (10 mg/L) in this case was comparable to that achieved in *E. coli*, but with the advantage of simplified downstream processes due to protein secretion in the streptomycete (Šnajder et al., 2019). Similarly, the *Streptomyces halstedii* phospholipase expression was approximately 60 and 30 times higher in *S. lividans* TK24 than in *E. coli* and *Pichia pastoris*, respectively (Tao et al., 2019). Sianidis et al. (2006) and Sinsereekul et al. (2010) reported that their attempts to express a xyloglucanase from *Jonesia* sp. and a cutinase from *Thermobifida* sp. failed, respectively, in *E. coli* and *B. subtilis*, and *E. coli* and *P. pastoris.* Finally, Díaz et al. (2004) produced in *S. lividans* JI66 a xylanase from *Aspergillus nidulans* with a yield 3- and 19-fold higher than in lactic bacteria and *Saccharomyces cerevisiae*, respectively. Despite these successes at laboratory level, we are indeed unaware of any further scaling up at industrial level of recombinant enzyme production from streptomycetes. We can suppose that this is probably due to an overall limited protein productivity in streptomycetes that rarely reaches the g/L production level usually required for industrial application. As reported in Table 1, only in the case of a chitinase (Nguyen-Thi and Doucet, 2016), the protein productivity was more than 1 g/L. These results point out the crucial need to overcome intrinsic bottlenecks in protein productivity in streptomycetes, by redesigning their regulatory networks and secretion pathways by system biology, as recently proposed by Kim et al. (2020).

In Table 1, 21 are the recombinant proteins curing human diseases (Figure 1A), including those for treating cancer (interleukin, interferon, Tumor Necrosis Factor Alpha-TNF-α), cardiovascular pathologies (streptokinase, hirudin), and metabolic or auto-immune disorders (glucagon, phenylalanine ammonia-lyase, tendamistat). Recently, *S. lividans* TK24 was used for producing an *Actinoallomurus* A8-sourced glutenase, a promising candidate for oral enzymatic management of gluten toxicity (Cavaletti et al., 2019). Streptomycetes were also used to express 8 ‘target’ proteins, as antigens from *Mycobacterium tuberculosis* (Vallin et al., 2006) or the α-integrin A-domain for screening ligands for treating inflammatory disorders (Ayadi et al., 2007), and few diagnostic proteins (2) as the T Cell receptor CD4 for diagnosis of HIV infection (Fornwald et al., 1993). Biopharmaceutical production of proteins in streptomycetes is generally acceptable to the Food and Drug Administration and European Medicine Agency since these bacteria have been used for decades in industrial manufacturing of antibiotics, immunomodulating and antitumor drugs, and nutraceuticals (Marinelli and Marcone, 2011). Additionally, these naturally soil-inhabiting bacteria are recognized as useful components of natural ecosystem and they are considered safer than other microorganisms for agricultural use (Berini et al., 2019). Interestingly, besides the proteins listed in Table 1, *S. lividans* was employed by Cangene Corporation (now part of Emergent BioSolutions) for the recombinant production of the macrophage-colony stimulating factor Leucotropin^™^, a therapeutic agent that successfully completed Phase III trials for treating Hodgkin’s and non-Hodgkin’s lymphoma (Vrancken and Anné, 2009). To our best knowledge, this is the only reported case of a therapeutic protein production in streptomycetes that reached the clinical phases.

Finally, Table 1 includes 7 proteins without any direct industrial/therapeutic application: they were produced in streptomycetes for studying biochemical/functional properties and/or mode of action, as in case of the novel N-substitute formamide deformylase from *Arthrobacter pascens* involved in the metabolism of isonitriles (Fukatsu et al., 2005). Another example is VanYn, a D,D-dipeptidase/D,D-carboxypeptidase identified as the sole resistant determinant in the glycopeptide producer *Nonomuraea gerenzanensis* (Binda et al., 2013; Dalmastri et al., 2016). VanYn expression in *Streptomyces venezuelae* allowed a higher production than in *E. coli* (Binda et al., 2012), and contributed to elucidating cell wall turnover during antibiotic production (Marcone et al., 2010a, 2014).

## Where Do Recombinant Proteins Expressed in Streptomycetes Come From?

71 of the proteins listed in Table 1 derive from prokaryotes and 23 from eukaryotes (Figure 1B). Most of prokaryote-sourced proteins come from Gram-positive bacteria: 49 are from *Streptomyces* or *Streptoverticillium* spp., or other actinomycetes as *Nonomuraea, Kitasatospora*, or *Thermobifida* spp. This is not surprising, as heterologous expression is facilitated when the host is phylogenetically related to the homologous producer, due to the similar metabolic and genetic background (Binda et al., 2013). Streptomycetes (DNA G + C > 60%) offer an optimized codon usage for high G + C content genes and they represent a complementary tool versus *E. coli* (DNA G + C *ca.* 51%). For instance, chitinases, usually produced by soil-inhabitant actinomycetes, were successfully produced in streptomycetes (Berini et al., 2019). Cloning a *S. coelicolor* chitinase in *S. lividans* 10–164 resulted in 486-fold production improvement compared to *E. coli*, allowing gram-scale production for converting crystalline chitin in *N*-acetylglucosamine (Nguyen-Thi and Doucet, 2016). 9 additional recombinant proteins derive from the firmicutes *Bacillus subtilis* and *Streptococcus equisimilis*, and other 9 from the Gram-negative *Escherichia*, *Thermus*, and *Pseudomonas* spp. (Figure 1B). The thermostable cellulase from the bacteroidetes *Rhodotermus marinus* (Hamed et al., 2017) and the archaeal thermozyme (pernisine) (Šnajder et al., 2019), described above, complete the list of the prokaryote proteins.

Streptomycetes were successfully used for expressing metagenome-sourced bacterial enzymes (Berini et al., 2017). 2 lipases from enriched fed-batch bioreactors (Meilleur et al., 2009; Côté and Shareck, 2010) and 1 chitinase (named 53D1) from agricultural soil (Berini et al., 2019) were produced in different *Streptomyces* strains. In case of 53D1, the protein was secreted (45 mg/L) into the culture broth by *S. coelicolor* A3(2), with a clear improvement over its expression in *E. coli*, where the protein was mostly accumulated as inactive into inclusion bodies (Cretoiu et al., 2015). Enough 53D1 was produced in the streptomycete to test its activity as biopesticide (Berini et al., 2019).

The heterogeneity of eukaryote sources of the recombinant proteins expressed in streptomycetes confirms their versatility (Table 1 and Figure 1B). The homologous producers of the eukaryote proteins listed in Table 1 span from filamentous fungi or yeasts (2), to invertebrates (insect, leech, and jellyfish; 3) or vertebrates (fish and mammals; 18). Notably, 14 human proteins were produced in these hosts. A chronological analysis indicates that eukaryote protein expression in streptomycetes was more frequent in the 1990s, becoming after that rarer. The last example of eukaryote protein produced in *S. lividans* TK24 dated back to 2012 (Lule et al., 2012). This is probably due to recent developments in using engineered yeasts, and mammalian and insect cell lines for manufacturing high-value eukaryote proteins, especially those requiring post-translational modifications (Hunter et al., 2019).

## Which Is the Best Promoter/Vector/Host System for Recombinant Protein Production in Streptomycetes?

*S. lividans* strains are by far the most frequently used heterologous hosts, employed for producing 91 proteins listed in Table 1. 31 proteins were expressed in the parental *S. lividans* 66 (also named JI66 or 1326), whereas 37 in its derivative TK24, which is a two-plasmid-free mutant carrying streptomycin resistance mutation (*str-6*, SLP2^–^, SLP3^–^) (Kieser et al., 2000) (Figure 1C). 1 additional protein was produced in TK64, carrying the same mutations as TK24 plus the *pro-2* mutation, and 1 in TK54, characterized by *his-2, leu-2*, and *spc-1* mutations. The use of *S. lividans* TK24 has the following advantages: (i) low level of extracellular protease activity, (ii) poorly active restriction-modification system of exogenous DNA, (iii) known biochemistry/genetic background due to its high similarity to the model organism *S. coelicolor* A3(2) (Daniels et al., 2018). Other *S. lividans* used as hosts were the plasmid-free mutants *S. lividans* TK23 (for 3 proteins), carrying spectinomycin resistance mutation (*spc-1*, SLP2^–^, SLP3^–^), and its derivative JT46 (2 proteins) mutated in *rec-46* gene to reduce inter-plasmid recombination (Kieser et al., 2000). 4 proteins were produced in *S. lividans* TK21, which lacks only SLP2 plasmid. *Ad hoc* constructed *S. lividans* hosts were derivatives of *S. lividans* 66 or TK24, as the pleiotropic mutant *S. lividans* 10–164 (Hurtubise et al., 1995) defective in cellobiose and xylobiose uptake and used for producing a metagenome-sourced lipase (Meilleur et al., 2009; Côté and Shareck, 2010), and *S. lividans galK*^–^ (galactokinase-deficient mutant) for the production of *E. coli* galactokinase (Lichenstein et al., 1988). *S. lividans* GSAL1, used for the production of a xylanase and a α-amylase, overexpresses the morphogene *ssgA*, which pleiotropically controls growth and cell division. *ssgA* overexpression markedly enhances septation in vegetative hyphae, leading to fragmented growth and to wider hyphae, a phenotype that apparently favors protein production and secretion (Sevillano et al., 2016). Other streptomycetes employed as hosts were *S. coelicolor* A3(2) and its derivative M145 (3 proteins), *Streptomyces griseus* (3), *S. rimosus* (3), *Streptomyces hygroscopicus* (3), *S. venezuelae* (2), and *Streptomyces avermitilis* (1) (Table 1 and Figure 1C). Although less frequently used than *S. lividans*, in certain cases these alternative streptomycetes permitted the production of proteins poorly or not at all expressed in *S. lividans* (Binda et al., 2013; Berini et al., 2019), thus indicating that expanding the range of streptomycete hosts might be promising.

As regards to vectors, the mostly used are high copy number replicative ones (in 93 cases) (Table 1 and Figure 1D) as for examples pIJ702 (25 proteins), pIJ486 (14), and pIJ86 (7 proteins). pIJ702 vector, which carries thiostrepton resistance (*tsrR*) and tyrosinase production (*mel*^+^) markers, is the derivative of pIJ350, a non-conjugative broad host range vector (Kieser et al., 2000). pIJ486 (*tsrR*) derived from pIJ101, which contains the promoterless *neo* gene (kanamycin resistance) and lacks both the transfer function and the *sti* locus that usually confers ‘strong incompatibility’. Removing the *sti* locus increases the chance that different plasmids can be retained at similar copy numbers (Deng et al., 1988; Kieser et al., 2000). The more recent pIJ86 carries apramycin resistance marker (*aprR*) and it is a conjugative vector used for the strong constitutive expression of proteins under erythromycin promoter (*ermE*^∗^ promoter) from *Saccharopolyspora erythraea*. Recent works (Sevillano et al., 2013, 2017) described new replicative high copy number ‘marker-free’ systems, which allowed the production of high levels of proteins without using antibiotics as selection markers. One example is based on the presence of a toxin gene localized in the genome and of an anti-toxin gene located on the expression plasmid of the *yefM/yoeBsl* operon from *S. lividans* (Sevillano et al., 2013). Only for 5 proteins, replicative moderate or low copy number vectors were used. For instance, the moderate copy number pIJ12739 was constructed for the expression of the phospholipase D from *S. halstedii* in *S. lividans* TK24, following the same approach previously described by Fernández-Martínez and Bibb (2014) to produce a dual-promoter expression vector (Tao et al., 2019). The low copy number pIJ943 was used for producing the 31-*O*-demethyl-FK506 methyltransferase in *S. lividans* (Motamedi et al., 1996). For only 3 proteins, integrative vectors were employed such as pAB04 – low copy number plasmid used for producing a phytase (Carrillo Rincón et al., 2018), or pIJ8600 – single copy number vector employed for the expression of the cutinase from the Gram-positive *Thermobifida* sp. in *S. rimosus* R7 (Sinsereekul et al., 2010). Although less explored, integrative vectors might present some advantages. When the integrative single copy number pHM8a plasmid was used for expressing a chitosanase, productivity was comparable to that achieved with replicative multicopy pFDES plasmid, but with the advantage of not requiring antibiotic addition for selection (Dubeau et al., 2011). Interestingly, this last work is the only one, among those cited in this mini-review, which allowed a direct comparison on the effect of different vectors on protein yield. Most of the studies were driven by an empirical case-by-case approach to optimize the tools for a specific protein production, making difficult to draw final conclusions on which is the preferable vector system to be used.

In 20 cases (out of 94), the heterologous protein genes were cloned under the control of their native promoters, but more frequently streptomycete (or other actinomycete) heterologous promoters were used (Table 1 and Figure 1C). The heterologous promoters can be constitutive (e.g., *vsi*p from *S. venezuelae*; *dag*p from *S. coelicolor; ermE^∗^*p from *Sac. erythraea; ssi*p from *Streptomyces albogriseolus; aph*p from *Streptomyces fradiae*) or inducible (e.g., *xysA*p from *S. halstedii*, induced by xylane; *pstS*p from *S. lividans*, by phosphate starvation and different carbon sources; *tcp830*p from *S. coelicolor*, by tetracycline*; tipA*p from *S. lividans*, by thiostrepton). Constitutive promoters were more frequently used than inducible ones (50 vs. 24 cases, respectively). If in *E. coli*, a balance between the vector copy number and the promoter strength is needed for controlling protein production and slowing down inclusion body formation (Adrio and Demain, 2014), in streptomycetes this problem is overcome by protein secretion. On the other hand, in streptomycetes, constitutive expression may cause a growth rate reduction negatively impacting on protein productivity: in these cases, inducible expression could be advantageous, although weak points of an inducible system remain as low level of expression, a narrow host range, and the need of an expensive inducer (Herai et al., 2004). As in the case of vectors, only very few studies systemically compared the effect of different promoters on protein yield. Sevillano et al. (2016) investigated the expression of a xylanase from *S. halstedii* cloning the gene under the control of six strong promoters, including two commonly used (*vsi*p and *ermE*^∗^p) and four recently identified. Two belonging to the last group (*xysA*p and *pstS*p) performed better than those considered the golden standards, confirming that there is room for developing new tools for improving protein expression in streptomycetes.

In 30 out of the 94 proteins, the presence of native signal peptides (SP) guaranteed secretion in the heterologous hosts, while in 2 cases proteins expressed with their native SP accumulated into the cytoplasm and in 1 case the enzyme was recovered from the cell wall fraction (Table 1). In streptomycetes, the Sec pathway constitutes the main secretion system (Anné et al., 2012). Accordingly, proteins to be secreted have N-terminal hydrophobic SP, followed by a longer hydrophobic H-domain and a C-terminal part containing at the end three amino acids which form the signal peptidase recognition site. Other minor secretion systems were reported, including the twin-arginine dependent translocation (Tat) pathway (Anné et al., 2012). The Tat machinery exports fully folded proteins across the cytoplasmic membrane: SPs that target proteins to this pathway resemble Sec SPs, but contain a conserved twin-arginine motif in the N-region (Valverde et al., 2018). A comparison between the efficiency of these two pathways for recombinant protein production showed that replacing Sec-dependent SP with Tat-dependent SP drastically reduced protein expression (Schaerlaekens et al., 2004). When native SPs were absent or not functional, heterologous genes were fused to SP encoding sequences from genes for highly expressed/secreted endogenous *Streptomyces* proteins (Anné et al., 2016), such as the one from the subtilisin inhibitor (*vsi*) of *S. venezuelae* CBS762.70 (Van Mellaert et al., 1998). Other SP sequences, frequently used in *Streptomyces* expression-systems are also listed in Table 1. They derived from the genes for the trypsin-like protease (*srT*) from *S. rimosus*, for the α-amylase from *Streptomyces tendae*, *S. griseus, S. lividans*, or *Streptomyces limoseus*, for the melanin operon gene (*melC1*) from *Streptomyces antibioticus*, for the subtilisin inhibitor (*ssi*) from *S. albogriseolus*. The final result is that in 77 out of the 94 proteins listed in Table 1, the recombinant proteins were completely secreted with productivities up to 100s of mg/L (Guan et al., 2015). In the few cases (8) where proteins were accumulated into cytoplasm, their productivity was generally low. 7 proteins were found produced either inside or outside the cells, whereas VanYn was localized in the cell wall fraction where it plays its physiological role in antibiotic resistance (Marcone et al., 2010a; Binda et al., 2012, 2013).

## Conclusion

From the analysis of the literature in the last four decades, it emerges that, although promising, streptomycetes have been used for heterologous protein production less than their potential indicates to do. Notwithstanding their efficient protein secretion machine – which definitively facilitates downstream operations and protein purification – the mycelial lifestyle of these bacteria has probably discouraged scientists to use them more frequently. In liquid media, streptomycetes grow as mycelial pellets consisting of cells in different physiological states, and cultures are not homogenous and might become very viscous. In this regard, combining different specific mutations as *ssgA* for improving disperse growth (Sevillano et al., 2016), and *galK* for generating auxotrophic mutants not requiring antibiotic-dependent selection (Lichenstein et al., 1988) might facilitate upstream processes. Additionally, formulation of novel cultivation media – replacing those used for antibiotic biosynthesis – could facilitate protein downstream (Binda et al., 2013; Berini et al., 2019). Another aspect probably limiting their application is that streptomycetes cannot be genetically manipulated by the methods commonly used for *E. coli* and *S. cerevisiae.* They need *ad hoc* protocols based on intergeneric conjugation or protoplast transformation (Kieser et al., 2000; Marcone et al., 2010b, c). With time, these protocols have become available and, as reported in this review, nowadays we can count on a large variety of vectors, promoters, and SP sequences. What is still missing is the systematic and critical comparison of the available toolkits. Optimization of protein production is still conducted following a case-by-case – and somehow random – approach. Finally, an important issue is the intrinsic low protein productivity of streptomycetes in comparison with the mostly used *E. coli* and yeasts. Further improvements, in this sense, are urgently needed and may derive from system and synthetic biology approaches, still poorly applied to streptomycetes. Indeed, progresses in system biology and -omics technologies may shed light on the interplay of elements involved in protein expression, thus helping in the rational improvement of both expression platforms and fermentation conditions, finalized at reducing the metabolic burden due to heterologous protein production. A demonstration is present in the pioneering work conducted by Muhamadali et al. (2015) on a *S. lividans* strain producing the murine TNF-α, where heterologous protein expression determined profound changes in the metabolomics of the streptomycete causing an overflow of organic acids and sugars. In post-genomic era, a further ambitious goal is applying synthetic biology approaches for building a *Streptomyces* ‘super host’ with metabolic networks rewired to facilitate heterologous protein expression. Synergic application of genome minimization strategies (i.e., systematic removal of those elements – as secondary metabolites or proteases – that can hamper protein production) and engineering of translation and transcription machineries, might help reaching this goal (Kim et al., 2020). To this end, it is encouraging considering that performing *Streptomyces* ‘super hosts’ have been already constructed for the heterologous production of antibiotics (Gomez-Escribano and Bibb, 2011; Myronovskyi et al., 2018). We believe that integrating these tools could help in improving streptomycetes as robust producers of recombinant proteins, increasing their competitiveness to other platforms and stimulating their large-scale application as cell factories.

## Author Contributions

FB and EB collected the data and analyzed them. FB, FM, and EB co-wrote the review. FB prepared the figure and the table. EB coordinated the work. All authors contributed to the article and approved the submitted version.

## Conflict of Interest

The authors declare that the research was conducted in the absence of any commercial or financial relationships that could be construed as a potential conflict of interest.
